# Association testing to detect gene–gene interactions on sex chromosomes in trio data

**DOI:** 10.3389/fgene.2013.00239

**Published:** 2013-11-13

**Authors:** Yeonok Lee, Debashis Ghosh, Yu Zhang

**Affiliations:** Department of Statistics, Penn State University, University ParkPA, USA

**Keywords:** binary traits, gene–gene interaction, generalized linear mixed effect model, logistic model, trio data, sex chromosomes

## Abstract

Autism Spectrum Disorder (ASD) occurs more often among males than females in a 4:1 ratio. Among theories used to explain the causes of ASD, the X chromosome and the Y chromosome theories attribute ASD to the X-linked mutation and the male-limited gene expressions on the Y chromosome, respectively. Despite the rationale of the theory, studies have failed to attribute the sex-biased ratio to the significant linkage or association on the regions of interest on X chromosome. We further study the gender biased ratio by examining the possible interaction effects between two genes in the sex chromosomes. We propose a logistic regression model with mixed effects to detect gene–gene interactions on sex chromosomes. We investigated the power and type I error rates of the approach for a range of minor allele frequencies and varying linkage disequilibrium between markers and QTLs. We also evaluated the robustness of the model to population stratification. We applied the model to a trio-family data set with an ASD affected male child to study gene–gene interactions on sex chromosomes.

## 1. Introduction

Autism Spectrum Disorders (ASDs) refer to a collection of developmental disabilities in social interaction, communication, and behavior. The prevalence of autism and related ASDs is increasing and about 1% of children need education and social care (Baird et al., 2006). A more recent study estimates that the worldwide median autism spectrum disorder prevalence is 62 out of 10,000 (Elsabbagh et al., 2012).

ASD is four times more common in males than in females (Chakrabarti and Fombonne, 2001). The bias could be in part due to the fact that females are less likely to be diagnosed as ASD at the equivalent level of autistic traits in males (Dworzynski et al., 2012). ASD is also environmental and genetic (Persico and Bourgeron, 2006; Matsuzaki et al., 2012). In Baron-Cohen et al. (2011), they summarized three possible factors that may attribute psychological and physiological changes in the male brain: (a) the masculinizing effect of fetal testosterone; (b) X- and Y-linked theories; (c) autosomal penetrance theory.

Intuitively, the sex chromosomes represent a reasonable starting point in order to find the causes of the gender bias in ASD. In fact, studies found that the X chromosome contains genes that are highly expressed in brain tissues compared to other tissues (Nguyen and Disteche, 2006). This supports the important role of the X chromosome in brain functions, which is also evident from the X-linked mental disabilities. There are also Y-linked male-specific genes expressed in human brains, such as *SRY, ZFY*, and *PCDH11R* (Mayer et al., 1998; Durand et al., 2006). The association studies of the genes on X or Y chromosomes with ASD include Serajee and AH (2009), Noor et al. (2010), Chung et al. (2011), Kaya et al. (2012). These studies focused on one of the sex chromosomes but not both at the same time.

For family-based association studies, the transmission/disequilibrium test [TDT, Spielman et al. (1993)], its generalizations such as the sib transmission/disequilibrium test [S-TDT, Spielman and Ewens (1998)], and the family-based association test [FBAT, Horvath et al. (2001)] are standard choices for qualitative data. However, they are not specifically designed for detecting interaction effects in genes. In this article, we look for interaction effects between two genes on sex chromosomes in males. No studies have been conducted to determine attribution of the diseases to gene–gene interactions on the sex chromosomes. We will seek to determine if there are gene–gene interactions on sex chromosomes that drive the gender bias in ASD.

Mixed effects models have been widely adopted in a wide range of disciplines. Mixed effects models use both fixed and random effects. Fixed effect parameters represent the average changes in the response variable, while random effects usually represent the subject-to-subject variability. Examples of the latter include batches in a chemical experiment, classrooms in an education setting, and members in a family. Recently, mixed effects models have received a significant attention in genetic association tests that account for the population stratification and the correlation among the individuals (Zhang et al., 2009; Wang et al., 2011; De Lobel et al., 2012; Zhou and Stephens, 2012). Zhou and Stephens (2012) developed a genome-wide efficient mixed-model association (GEMMA), in which the related polygenic effects are treated as a random effect. De Lobel et al. (2012) introduced a mixed effects model that incorporates gene–gene interactions in autosomal chromosomes. These mixed effects models for the association study, with or without gene–gene interaction, are currently designed for quantitative response variables only.

In this article, we apply a generalized linear mixed effects model to handle dichotomous responses and genetic interaction effects. Generalized mixed effects models (Breslow and Clayton, 1993; McCulloch, 1997) are the extensions of generalized linear models (Nelder and Wedderburn, 1972; McCullagh and Nelder, 1989). Generalized linear models are regression models for different response types and the expected value of the response μ_*i*_ is

$$\mu_{i}=g^{-1}\left(x_{i}\beta \right),$$

where *g* is an invertible link function, *x*_*i*_ is the *i*th observation for fixed effects, and β is fixed effect coefficients. In a generalized linear mixed effects model with two random effects *A* and *E*, the expected response is

$$\mu_{i}=g^{-1}\left(x_{i}\beta +A_{i}+E_{i}\right).$$

For a binary response, the logistic link is defined as *g*(μ_*i*_) = log(μ_*i*_/(1 − μ_*i*_)) and

$$\mu_{i}=1/(1+\exp\left(-\left(x_{i}\beta +A_{i}+E_{i}\right)\right)).$$

Here, μ_*i*_ can be the trait probability for the *i*th individual. In our study, we treat genetic effects, including an interaction effect, as fixed effects, but we further include unlinked autosomal effects as random effects.

## 2. Materials and methods

### 2.1. The model

We introduce a generalized mixed effects model for the association test on sex chromosome in males. Family-based trio data with an affected son will be considered. Assume that two unlinked additive QTLs are associated with the disease. Our model is written as

(1) $$\log\left(\frac{p_{ij}}{1-p_{ij}}\right)=\beta_{0}+\beta_{1}X_{ij}+\beta_{2}Y_{ij}+\beta_{12}XY_{ij}+A_{ij}+E_{ij},$$

where *p*_*ij*_ is the trait probability for the *j*th individual in the *i*th family, β_0_, β_1_, β_2_, and β_12_ are the regression coefficients, *X*_*ij*_ and *Y*_*ij*_ denote the genotypes at two loci of the *j*th individual in the *i*th family and they are either 0 or 2, and *XY*_*ij*_ is the interaction effect of the two. Let *A*_*ij*_ be the random effect due to the unlinked autosomal QTLs and *E*_*ij*_ be the environmental random effect. The variance - covariance matrix of the two random effects between the *j*th and the *k*th individuals in the *i*th family is given by

(2) $$\Omega_{ijk}=\{\begin{array}{ll} \sigma_{a}^{2}+\sigma_{e}^{2} & \text{if }j=k\\ \phi_{ijk}\sigma_{a}^{2}+\sigma_{e}^{2} & \text{if }j\neq k, \end{array}$$

where ϕ_*ijk*_ is twice of the kinship coefficient between the *j*th and the *k*th individual in the *i*th family. We assume that the random effects follow a Normal distribution with mean 0 and variance Ω_*ijk*_. The analysis is conducted using an R package pedigreemm (Vazquez et al., 2010). When we consider a model with a binary response variable and random effects, the full maximum likelihood analysis requires a numerical integration technique. In such case, the package pedigreemm uses the Laplace approximation (Tierney and Kadane, 1986). The fixed effects are estimated based on the *iterative re-weighted least squares algorithm* (Green, 1987). Under the assumption that the estimates follow a Normal distribution, pedigreemm generates the test statistics *z* = $\hat{\beta }$/*s.e*($\hat{\beta }$) and the corresponding p-values (for two-sided test) under the null hypothesis of no association. Our study results are based on these outputs.

### 2.2. Simulation study

Assume that the two QTLs are unlinked. We generated two markers in linkage disequilibrium (LD) with the two QTLs from *D*′ = 0 to *D*′ = 1 with an increment of 0.1. For *D*′ = 0, the markers have no LD with QTLs and therefore have no association with the disease. For *D*′ = 1, the markers have complete dependency to QTLs. We assume that QTLs and markers have the same minor allele frequencies (MAFs), which we vary in the simulations at 0.1, 0.3, and 0.5. We set the sample size to be 2000 (1000 families with father and one son in each family) and use σ^2^_*a*_ = 0.5 and σ^2^_*e*_ = 1.

First, we explain how the correlated random effects within a family are generated. Similarly to De Lobel et al. (2012), the random effects due to unlinked autosomal QTLs are generated as follows:

$$\begin{array}{l} A_{iF}\sim N(0,\sigma_{a}^{2})\\ A_{iO}=0.5A_{iF}+\sqrt{0.75}v,\text{where }v\sim N\left(0,\sigma_{a}^{2}\right), \end{array}$$

where *A*_*iF*_ and *A*_*iO*_ are unlinked autosomal random effects of a father and a son, respectively, in the *i*th family. This leads to the correlation between *A*_*iF*_ and *A*_*iO*_ at 0.5 · σ^2^_*a*_.

Second, we need to generate family samples in which the father is unaffected and the son is affected. Initially, we generated a large enough number of samples and selected family samples in which the father is unaffected and the son is affected. The website of the R code for this sampling method is provided in the Supplemental data. The R code generates genotypes with specified MAFs and the two random effects (including *A*_*ij*_ as explained above), obtains binary responses based on the trait probabilities, and returns family samples that contain an unaffected father and an affected son.

When the samples are selected under such condition, the sample means of the random effects *A*_*iF*_ and *A*_*iO*_ can be shifted away from zero. In simulation study, the random effect samples follow a Normal distribution but the two sample means are not equal to zero, which violates the model assumption about the random effect having mean zero. We tested if the discrepancy between sample means and zero affects p-values for testing the significance of interaction effect. We simulated 100 datasets using the parameters in the simulation studies (Tables 1, 2) and found that the discrepancy in means did not affect *p*-value of the interaction effect (data not shown).

**Table 1:** **MAFs of QTLs for simulation study when no population stratification is present**.

	**MAFs**					
MAF of X chromosome	0.1	0.3	0.5	0.1	0.1	0.3
MAF of Y chromosome	0.1	0.3	0.5	0.3	0.5	0.5

**Table 2:** **Coefficients for simulation study**.

	**β_0_**	**β_1_**	**β_2_**	**β_12_**	**Additional risk**
coef A	0	0	0	0.5	0.38
coef B	0	0.25	0.25	0.75	0.25
coef C	0	0.5	0.5	0.5	0.1
coef D	0	0.75	0.75	0.25	0.03

*Additional risk on the disease due to the interaction effects is exp(2β_1_ + 2β_2_ + 4β_12_)/(1 + exp(2β_1_ + 2β_2_ + 4β_12_) - exp (2β_1_ + 2β_2_)/(1 + exp(2β_1_ + 2β_2_))*.

#### 2.2.1. No population stratification

We conducted 24 simulation studies with varying MAFs and regression coefficients β_0_, β_1_, β_2_, and β_12_. They are summarized in Tables 1, 2, respectively. The regression coefficients in Table 2 represent the following: coef A (β_0_ = 0, β_1_ = 0, β_2_ = 0, and β_12_ = 0.5) has no main effect but an interaction effect, coef B (β_0_ = 0, β_1_ = 0.25, β_2_ = 0.25, and β_12_ = 0.75) has a larger interaction effect, coef C (β_0_ = 0, β_1_ = 0.5, β_2_ = 0.5, and β_12_ = 0.5) has equal main and interaction effects, and coef D (β_0_ = 0, β_1_ = 0.75, β_2_ = 0.75, and β_12_ = 0.25) has larger main effects. We note that the same magnitude of the interaction coefficient does not reflect the same amount of contribution to the disease risk. Despite the fact that coef A and coef C have the same interaction coefficient β_12_ = 0.5, the increase in risk by adding the interaction effect are about 0.38 and 0.1, respectively. This is due to the fact that the interaction effect occurs on top of the two main effects. When the two main effects are smaller, the increase in disease risk due to the interaction effect becomes larger.

#### 2.2.2. Population stratification

It has been known that population stratification can result in spurious association findings in mixed effects model settings (Abecasis et al., 2000). De Lobel et al. (2012) orthogonalized the genotype scores into within and between-family effects in order to avoid such spurious findings. We study the impact of population stratification on the type I error and power of detecting gene–gene interaction effects using the proposed model.

We consider two populations, each with 1000 individuals. The population stratification can be formulated in three ways in Model (1):

- Different β_0_: the disease prevalence is different due to other factors
- Different MAFs: MAFs can be different in two populations
- Different β_1_, β_2_ and β_12_: the genotype effects can be different

We considered two scenarios: case 1 includes the first two conditions but not the third condition and case 2 includes all three conditions. For case 1, we used β_0_ = 0 for Population 1 and β_0_ = 0.2 for Population 2 and the three combinations of MAFs in Table 3. In Population 1, the MAFs of X and Y chromosomes are the same while they are not identical (0.5 and 0.3) in Population 2. The coefficients β_1_, β_2_, and β_12_ are set to be the same for the two populations. For case 2, while keeping the first two conditions the same as those in case 1, we use the regression coefficients from Table 2 for Population 1 and use 0.2 for all the regression coefficients β_0_, β_1_, β_2_, and β_12_ for Population 2.

**Table 3:** **MAFs of QTLs for simulation study when population stratification is present**.

**Population 1**		**Population 2**	
MAF of X chr	MAF of Y chr	MAF of X chr	MAF of Y chr
0.1	0.1	0.5	0.3
0.3	0.3	0.5	0.3
0.4	0.4	0.5	0.3

### 2.3. Application to autism study

We obtained the parent-offsprings trios data from dbGaP at http://www/ncbi.nlm.nih.gov/gap through dbGaP accession number phs000267.v1.p1. As the interest is on the interaction of genetic variables on the X and Y chromosomes, we selected families who have an affected son. We have a total of 2216 individuals in 1108 families. We focused on 90 and 2 SNPs on the X and Y chromosomes, respectively. The ninety SNPs on the X chromosome are in *PTCHD1, TBL1X*, and *NLGN3*, which are candidate genes for autism spectrum disorder (Noor et al., 2010; Chung et al., 2011; Kaya et al., 2012). The two SNPs on Y chromosome are based on Serajee and AH (2009). Out of the six SNPs in Serajee and AH (2009), only three (*rs9306845, rs9786893*, and *rs16980459*) are available in dbGaP data. Also, the two genotypes *rs9786893* and *rs16980459* are identical and hence only two are used in the study. In total 180 tests were conducted. We excluded families whose SNP is missing, and thus the number of families we used for testing varies. The smallest number of families we used is 1077.

## 3. Results

### 3.1. Simulation study

#### 3.1.1. No population stratification

**Figure 1:**
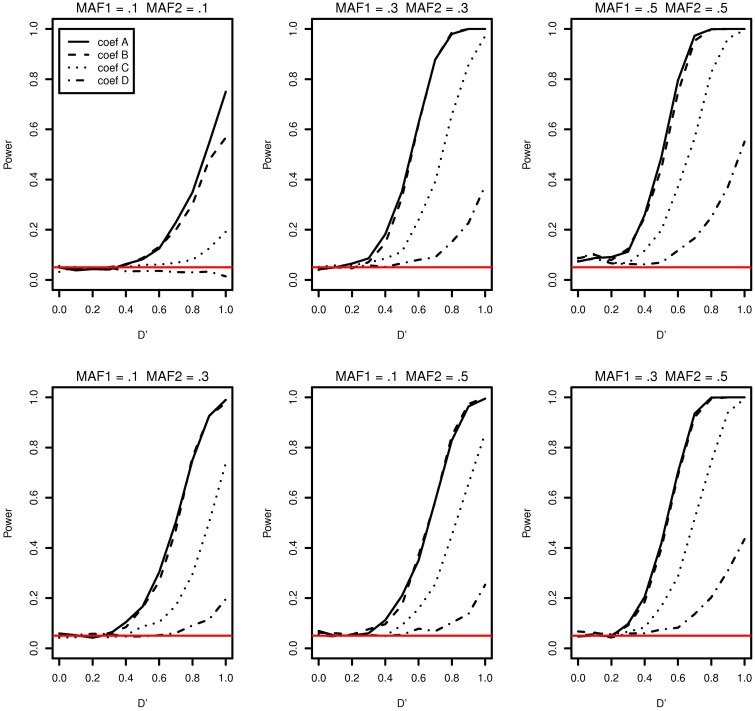
**Type I error and power of detecting interaction effects with different MAFs and four regression coefficients**. For all four β_0_ = 0, coef A: β_1_ = 0, β_2_ = 0, and β_12_ = 0.5, coef B: β_1_ = 0.25, β_2_ = 0.25, and β_12_ = 0.75, coef C: β_1_ = 0.5, β_2_ = 0.5, and β_12_ = 0.5, coef D: β_1_ = 0.75, β_2_ = 0.75, and β_12_ = 0.25, and σ^2^_*a*_ = 0.5 and σ^2^_*e*_ = 1, when the sample size is 2000 (1000 families). The red horizontal line indicates the 0.05 significance level.

The type I error and power of detecting interaction effects for varying LD, MAFs and regression coefficients are summarized in Figure 1. The MAFs in the top row of Figure 1 are equal in both markers while MAFs are different in the bottom row. Each plot includes the results of four different regression coefficients listed in Table 2 varying D′ from 0 to 1.

The power of detecting interaction effects is significantly affected by the MAF values. This can be best shown by comparing the first and the last plots in the top row. When MAFs are 0.1 on two markers, the maximum power to detect the interaction effect is 0.75 for coef A when LD is equal to 1. Meanwhile, when MAFs are both 0.5, the power is about 0.8 for coef A when LD is larger than 0.6. The power is associated with the number of nonzero interaction genotypes. The expected number of nonzero genotypes in the interaction is only 20 when both MAFs are 0.1, which is 1% of the sample size 2000. This may not be large enough number to be able to detect the interaction effects at the power level 0.8.

Within each plot, it is apparent that the power increases as the additional risk due to the interaction effect increases (see the last column in Table 2). The power of detecting interaction effects is generally 0.8 or larger when *D*′ is 1 in most of the cases, excluding coef D in all plots and all regression coefficients combinations in MAF1 = 0.1 and MAF2 = 0.1. The proposed model detects interaction effects at a power greater than 0.8 (*D*′ = 1) when their risk is as low as 0.1 with MAFs larger than 0.1.

The power in general increases as *D*′ increases. However, there are two exceptions; first, when coef D (MAF1 = 0.1 and MAF2 = 0.1) and when *D*′ is less than 0.4 (MAF1 = 0.5 and MAF2 = 0.5). In the former, both the number of nonzero genotypes in interaction effect and the additional risk due to interaction effect are not large enough to be detected by the proposed model. When the sample size is 5000, the decreasing trend in power disappears, although the power is still as low as 0.1 at *D*′ = 1 (See Supplementary Data: Supplemental Figure 1). In the latter, the type I errors in the third plot (MAF1 = 0.5 and MAF2 = 0.5) in Figure 1 are 0.073, 0.086, 0.088, 0.075 for coef A to coef D, respectively, and they are larger than expected. The 95% confidence intervals are (0.057, 0.089), (0.068,0.104), (0.070, 0.106), and (0.058, 0.092), respectively. These slightly inflated type I errors do not seem to appear when the sample size is 5000, in which type I errors are 0.054, 0.064, 0.078, and 0.063, respectively. The type I error for coef C is the only one that is statistically significantly larger than expected. The 95% confidence interval is (0.061,0.0950). We suspect that these two exceptional trends are due to the lack of information by relatively small sample size.

Testing interaction effects is reliable using the proposed model excluding some cases when the additional risk due to interaction effects are insignificant or when the number of nonzero interaction genotypes is quite small. On the other hand, we found that the variance estimates, ${\hat{\sigma }}_{a}^{2}$ and ${\hat{\sigma }}_{e}^{2}$ in Equation (2), are heavily biased and close to zero in the simulation studies. Consequently, the fixed effect estimates are identical or close to those of a logistic regression model in the simulation study settings. While the generalized linear mixed effects model is computationally more demanding, it can accommodate more general family structures.

**Figure 2:**
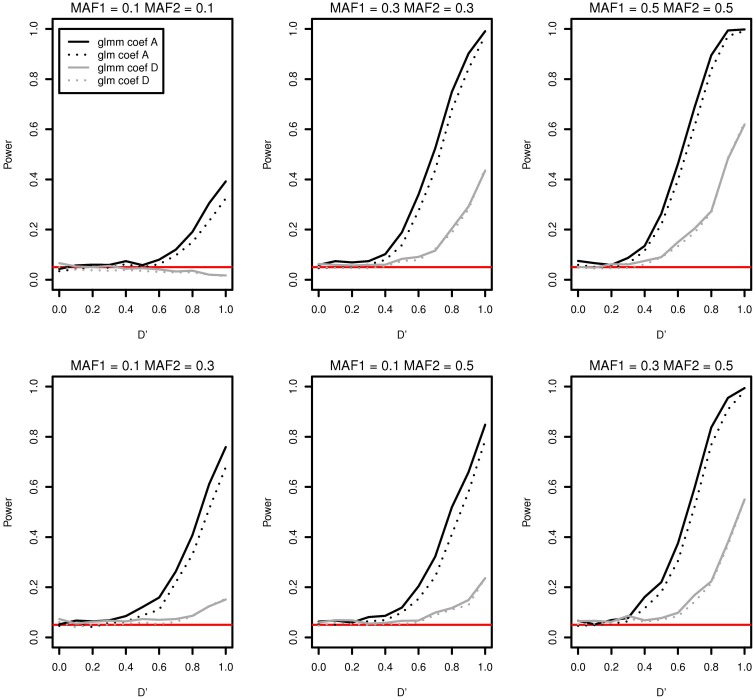
**Type I error and power of detecting interaction effects with different MAFs using a generalized linear mixed effects model (solid) and a generalized linear model (dotted) when samples with five family members are randomly generated**. Two regression coefficients are β_0_ = 0, coef A: β_1_ = 0, β_2_ = 0, and β_12_ = 0.5 (shown in black), coef D: β_1_ = 0.75, β_2_ = 0.75, and β_12_ = 0.25 (shown in gray), σ^2^_*a*_ = 10 and σ^2^_*e*_ = 0.01, and the sample size is 2000 (400 families). The red horizontal line indicates the 0.05 significance level.

As an example, we compared the type I error and the power of the two models in Figure 2 when there are a father and four male siblings in a family. Figure 2 contains only two cases coef A and coef D for a demonstration. There is no significant differences in power between two models for coef D (gray curves). The two black plots in Figure 2 are well separated and this shows that the generalized linear mixed effects model performs better in detecting interaction effects for coef A for all MAFs. The discrepancy in power for coef A at *D*′ = 1 is as high as 0.080 in MAF1 = 0.3 and MAF2 = 0.3. In addition, the generalized linear mixed effects model is more suitable to incorporate the genetic correlation among family members even if it is computationally more expensive.

Breslow and Clayton (1993) illustrated that generalized mixed effects model estimates are biased when applied to clustered binary data, and therefore we anticipated that the model estimates would be biased. Table 4 shows the median interaction effect estimates and the 95% confidence intervals at *D*′ = 0, 0.5, and 1 when the two MAFs are equal to each other. As expected, the estimated parameters are underestimated in all cases. Regardless, the power of detecting interaction effects is larger than 0.8 at *D*′ ≥ 0.8 excluding when coef D and when MAFs are both 0.1. See Figure 1. Based on the performance of detecting interaction effects, the proposed model is suitable to detect interaction effects in the simulation study settings.

**Table 4:** **The median and 95% confidence interval of the interaction coefficient estimates at *D*′ = 0, 0.5, and 1 when the two MAFs are the same**.

**MAFs**	**D′**	**coef A**		**coef B**		**coef C**		**coef D**	
		**Med**.	**CI**	**Med**.	**CI**	**Med**.	**CI**	**Med**.	**CI**
	0	−0.01	(−0.23, 0.20)	−0.00	(−0.21, 0.22)	−0.01	(−0.22, 0.20)	−0.00	(−0.23, 0.22)
0.1	0.5	0.09	(−0.14, 0.32)	0.08	(−0.15, 0.33)	0.04	(−0.18, 0.29)	0.02	(−0.20, 0.26)
	1	0.39	(0.15, 0.72)	0.51	(0.20, 3.82)	0.31	(0.03, 3.51)	0.13	(−0.14, 3.34)
	0	−0.00	(−0.09, 0.08)	−0.00	(−0.09, 0.09)	0.00	(−0.09, 0.09)	−0.00	(−0.08, 0.08)
0.3	0.5	0.08	(−0.00, 0.18)	0.09	(−0.01, 0.17)	0.05	(−0.05, 0.14)	0.02	(−0.08, 0.11)
	1	0.37	(0.28, 0.47)	0.43	(0.30, 0.57)	0.24	(0.14, 0.35)	0.10	(0.00, 0.20)
	0	−0.00	(−0.07, 0.07)	−0.00	(−0.07, 0.08)	−0.00	(−0.08, 0.07)	−0.00	(−0.07, 0.07)
0.5	0.5	0.08	(0.01, 0.16)	0.08	(0.01, 0.16)	0.05	(−0.03, 0.13)	0.02	(−0.06, 0.09)
	1	0.36	(0.27, 0.43)	0.37	(0.28, 0.45)	0.22	(0.14, 0.30)	0.10	(0.02, 0.18)
Coef.		0.5		0.75		0.5		0.25	

*Med. refers to the median interaction effect estimate, CI refers to the 95% confidence interval (2.5th percentile, 97.5th percentile), and Coef. refers to the true interaction effect coefficient*.

#### 3.1.2. Population stratification

**Figure 3:**
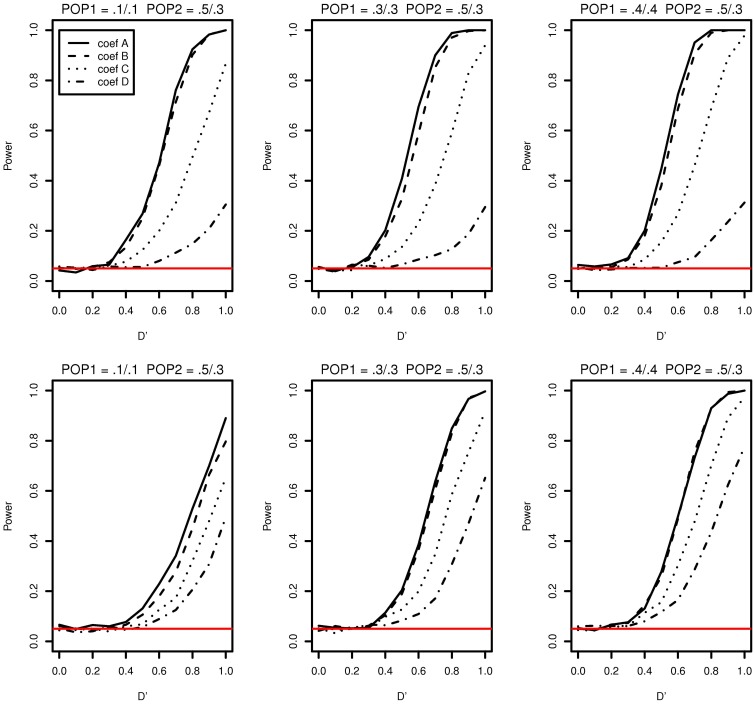
**Type I error and power of detecting interaction effects with different MAFs when population stratification is present with the equal sample size 1000 (500 families) in two populations**. Plots in the top row: β_0_ = 0 for Population 1 and β_0_ = 0.2 for Population 2 sharing the same regression coefficients coef A: β_1_ = 0, β_2_ = 0, and β_12_ = 0.5, coef B: β_1_ = 0.25, β_2_ = 0.25, and β_12_ = 0.75, coef C: β_1_ = 0.5, β_2_ = 0.5, and β_12_ = 0.5, coef D: β_1_ = 0.75, β_2_ = 0.75, and β_12_ = 0.25, and σ^2^_*a*_ = 0.5 and σ^2^_*e*_ = 1. Plots in the bottom row: β_0_ = 0 for Population 1 with the regression coefficients as above and β_0_ = β_1_ = β_2_ = β_12_ = 0.2 for Population 2, and σ^2^_*a*_ = 0.5 and σ^2^_*e*_ = 1 for both populations. The red horizontal line indicates the 0.05 significance level.

The simulation study results of type I error and power of detecting interaction effects when population stratification is present are shown in Figure 3. The plots in the top row correspond to case 1 and the ones in the bottom correspond to case 2. In both cases, the type I error rates are consistent around 0.05 and no spurious false positives are found. In both case 1 and case 2, the increase in power is mainly affected by the increase in MAFs: the larger MAFs the better power among the plots in both top and bottom rows. As explained earlier, this is related to the expected numbers of nonzero interaction genotypes. They are 160 [(0.1 × 0.1 + 0.5 × 0.3)/2 × 2000], 240, and 310, for the first, the second and the third columns, respectively in Figure 3.

If it were not known that there is population stratification, the first simulation setting (MAF1 = 0.1 and MAF2 = 0.1 in Population 1 and MAF1 = 0.5 and MAF2 = 0.3 in Population 2) can be considered as MAF1 = 0.3 and MAF2 = 0.2 with no population stratification. And this is equivalent to MAF1 = 0.2 and MAF2 = 0.3. Any loss in the power due to population stratification in case 1 can be revealed by comparing the power with the same MAFs with no population stratification. The type I error and the power of the first plot in Figure 3 can be compared with those in the first in the bottom (MAF1 = 0.1 and MAF2 = 0.3) and the middle one in the top (MAF1 = 0.3 and MAF2 = 0.3) in Figure 1. The power lies in the middle of the increasing trend as MAFs increase. Likewise, the middle plot in the top row in Figure 3, considered equivalent to MAF1 = 0.3 and MAF2 = 0.4, can be compared to the middle on the top (MAF1 = 0.3 and MAF2 = 0.3) and the last (MAF1 = 0.3 and MAF2 = 0.5) in Figure 1. This also does not indicate any loss in power due to population stratification with different MAFs in two populations.

The possible loss in the power when the main and interaction effects on the disease are different in two populations can be discovered by comparing powers in the top and ones in the bottom in Figure 3. In the top row of Figure 3, the main and interaction effects in two populations vary simultaneously as in Table 2, and in the bottom row, we replaced the regression coefficients to β_0_ = β_1_ = β_2_ = β_12_ = 0.2 in Population 2. In Population 2, the additional risk caused by the interaction effect is 0.1271, which is smaller than those in coef A and coef B and larger than coef C and coef D in Table 2. This is reflected in a decrease in power for coef A, coef B, and an increase in power for coef D, when we compare plots in the top and the ones in the bottom in Figure 3. For coef C, the change in power due to the different genetic effects seems to be affected by MAFs: the power decreases in the first plot in the bottom but stays constant in the second and the third plots, relative to the ones in the top in Figure 3.

In both case 1 and case 2, there is no spurious interaction effect detected. The impact of population stratification on the power of detecting the interaction effect using Model (1) has not found in the simulation study in neither case 1 nor case 2.

### 3.2. Application to autism study

Our interest lies in the presence of the interaction between two SNPs and the statistical test will be focused only on the interaction effects. The chance of falsely rejecting the null hypothesis (type I error) becomes larger when we conduct multiple statistical tests simultaneously for a given significance level α. The Bonferroni correction is a simple but conservative approach to correct this. Instead, we used a permutation test in order to find an adjusted type I error. In general, all the variables are permuted together to generate null datasets. In our case, however, family members share the same value in Y chromosome. In other words, the father and the son in a family have the same genotype on Y chromosome. Due to this constraints, we permute the two genetic variables separately. The fathers' genotype scores are permuted and the offsprings are given the same value of the father's. After 1000 repetitions, we found the adjusted significance level α' = 0.001 such that less than or equal to 50 (out of 1000) repetitions include at least one statistically significant interaction effect. Using α' = 0.001, we found no statistically significant interaction effect. The two SNPs on X chromosome, *rs2681644* and *rs2238860* that have the smallest p-values are shown on Table 5. Both are located on the intron region of *TBL1X*.

**Table 5:** **Two SNPs on X chromosome that show the smallest *p*-values**.

**X chromosome**		**Y chromosome**		**Three fixed effects**		**Four fixed effects**	
**SNP**	**Gene**	**SNP**	**Gene**	$\hat{\beta }$_12_	***p*-value**	$\hat{\beta }$_12_	***p*-value**
rs2681644	*TBL1X*	rs9306845	*TBL1Y*	0.2073	0.0066	0.19085	0.0139
rs2681644	*TBL1X*	rs9786893	*NLGN4Y*	0.2090	0.0081	0.19135	0.0174
rs2238860	*TBL1X*	rs9306845	*TBL1Y*	0.1888	0.0150	0.19423	0.0142
rs2238860	*TBL1X*	rs9786893	*NLGN4Y*	0.2260	0.0050	0.23351	0.0046

*Three fixed effects model is $\log(\frac{p_{ij}}{1-p_{ij}})=\beta_{0}+\beta_{1}X_{ij}+\beta_{2}Y_{ij}+\beta_{12}XY_{ij}+A_{ij}+E_{ij}$ and four fixed effects model is $\log(\frac{p_{ij}}{1-p_{ij}})=\beta_{0}+\beta_{1}X_{ij}+\beta_{2}Y_{ij}+\beta_{12}XY_{ij}+CNV_{ij}+A_{ij}+E_{ij}$ where CNV_ij_ is the copy number variance estimates on X chromosome for the jth member in the ith family*.

An advantage of regression models over a contingency table is the capability to include additional variables of information flexibly. We added an additional information on copy number variation (CNV) on X chromosome to the logistic regression mixed effects model. The additional variable CNV is obtained after processing the raw CNVs using an R package DNAcopy version 1.30.0 (Olshen et al., 2004). In this study, the variable CNV on the genes of our interest do not statistically significantly affect on the autism risk. The regression coefficients of interaction when CNV is included are given in the last two columns in Table 5.

## 4. Discussion

We applied a logistic regression model with mixed effects to detect gene–gene interactions on the sex chromosomes in trio data. Especially, only males who have both X and Y chromosomes are included for the study. In our study, we used binary response and explanatory variables and associated the potential correlation among family members using random effects. From the simulation studies, we find that the proposed model detected interaction effects at a power greater than 0.8 (*D*′ = 1) when the risk due to them is as low as 0.1 and MAFs are larger than 0.1. It is robust to population stratification and there is no increase in type I error rate.

**Figure 4:**
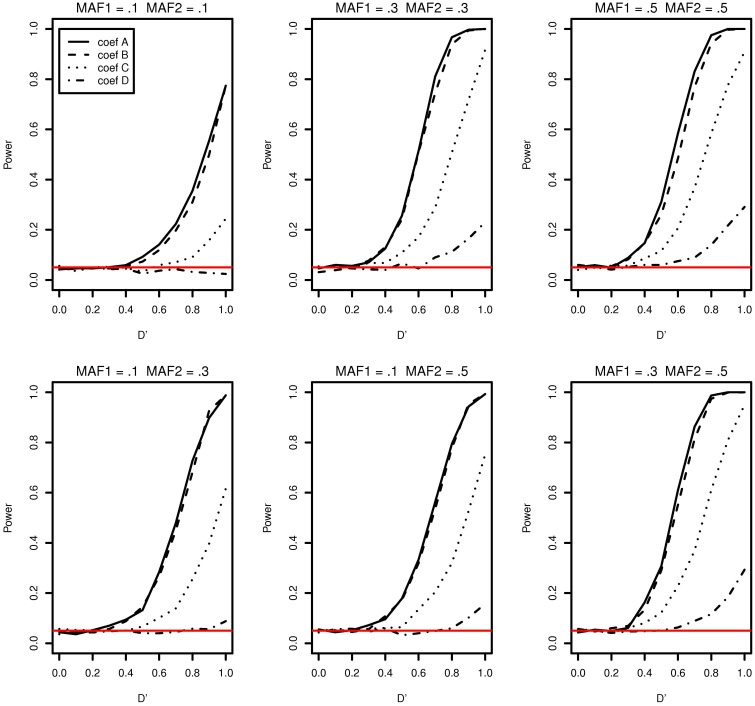
**Type I error and power of detecting interaction effects with different MAFs and four regression coefficients using an 1-1 matched logistic regression model with no population stratification**. For all four β_0_ = 0, coef A: β_1_ = 0, β_2_ = 0, and β_12_ = 0.5, coef B: β_1_ = 0.25, β_2_ = 0.25, and β_12_ = 0.75, coef C: β_1_ = 0.5, β_2_ = 0.5, and β_12_ = 0.5, coef D: β_1_ = 0.75, β_2_ = 0.75, and β_12_ = 0.25, and σ^2^_*a*_ = 0.5 and σ^2^_*e*_ = 1, and the sample size is 2000 (1000 families). The red horizontal line indicates the 0.05 significance level.

Family-based association study data consist of families in which at least a member is affected. Logistic regression models were studied for such data using conditional likelihood on all other possible cases that the specific number of family members are affected. This is referred to as ascertainment adjustment (Burton et al., 2000). In our case, a father is considered as a control and a son as a case in a family. Regarding this as one control and one case study in a family, referred to as 1-1 matched, the conditional likelihood estimate can be obtained by setting the intercept equal to 0, the new variables defined as X^*^_i_ = *X*_*iO*_ − *X*_*iF*_, and all the response variable set to 1 (Hosmer and Lemeshow, 1989 Chapter 7). A brief explanation on the background is provided in the Supplemental data. The type I error and power of the 1-1 matched logistic model are presented in Figure 4 for the MAFs and regression coefficients used in the no population stratification simulation study. The 1-1 matched logistic model performs slightly better in detecting the existing interaction effects when both MAF1 and MAF2 are 0.1. However, the logistic regression model with mixed effects performs better in power in all remaining cases. We suspect that the reason is in that the 1-1 matched logistic regression model infers the interaction effect of the population while the logistic model with mixed effect infers the interaction effects within the samples.

While the proposed model works well for detecting interaction effects and performs better in power compared to the 1-1 matched logistic regression model in the most of our simulation settings, the model underestimates the interaction effects. Therefore, the logistic regression model with mixed effects is not appropriate when the interest is in the parameter estimates or the true disease risks due to interaction effects.

## Author contributions

Yeonok Lee, Debashis Ghosh, and Yu Zhang designed the project. Yeonok Lee performed the data analysis and wrote the paper; Debashis Ghosh and Yu Zhang read and approved the final manuscript.

### Conflict of interest statement

The authors declare that the research was conducted in the absence of any commercial or financial relationships that could be construed as a potential conflict of interest.
