# Supplementary material for: Association testing to detect gene–gene interactions on sex chromosomes in trio data
Source: Front Genet. 2013 Nov 13;4:239. doi: 10.3389/fgene.2013.00239 (PMC3826485; doi:10.3389/fgene.2013.00239)
Supplement: Supplementary file 1 [file Presentation1.PDF]

SUPPLEMENTAL DATA

TYPE I ERROR AND POWER WHEN USING LOGISTIC REGRESSION FOR 1-1 MATCHED DATA

Consider a logistic regerssion model:

$$\log(\frac{p_{ij}}{1-p_{ij}}) = \beta' X_{ij},$$

where p_{ij} is the probability of the positive occurance of the jth member in ith family, $\beta = (\beta_0, \cdots, \beta_p)'$, and X_{ij} is a genotype vector of the jth member in the ith family. Note that the likelihood of the coefficient β for an individual k be

$$l_k(\beta) = y_k \pi(X_k) + (1 - y_k)(1 - \pi(X_k)),$$

where $\pi(X_k) = \exp(\beta' X_k)/(1 + \exp(\beta' X_k))$, with $\beta = (\beta_0, \cdots, \beta_p)$. Consider a 1-1 matched data, in which one member of a family is affected and another is not. The conditional likelihood of the ith family is

$$l_i(\beta) = \frac{(1 - \pi(X_{iF}))\pi(X_{iO})}{\pi(X_{iF})(1 - \pi(X_{iO})) + (1 - \pi(X_{iF}))\pi(X_{iO})}.$$

Simplifying this,

$$l_i(\beta) = \frac{\exp(\beta' X_{iO})}{\exp(\beta' X_{iF}) + \exp(\beta' X_{iO})}.$$

When we divide both denominator and nominator by $\exp(\beta' X_{iF})$, it is equivalent to a logistic regression with a new variable $X_{iS} - X_{iF}$ with no intercept and the response variables 1.

TYPE I ERROR AND POWER WHEN USING SAMPLE SIZE 5000

Supplemental Figure1 shows the type I error and power with the sample size 5000 using the MAFs are the regression coefficients in **Table1** and **Table2** in the main text.

R CODES

The sampling algorithm used for the model is available in `https://sites.google.com/site/yonoklee/glmm_epistatic`.

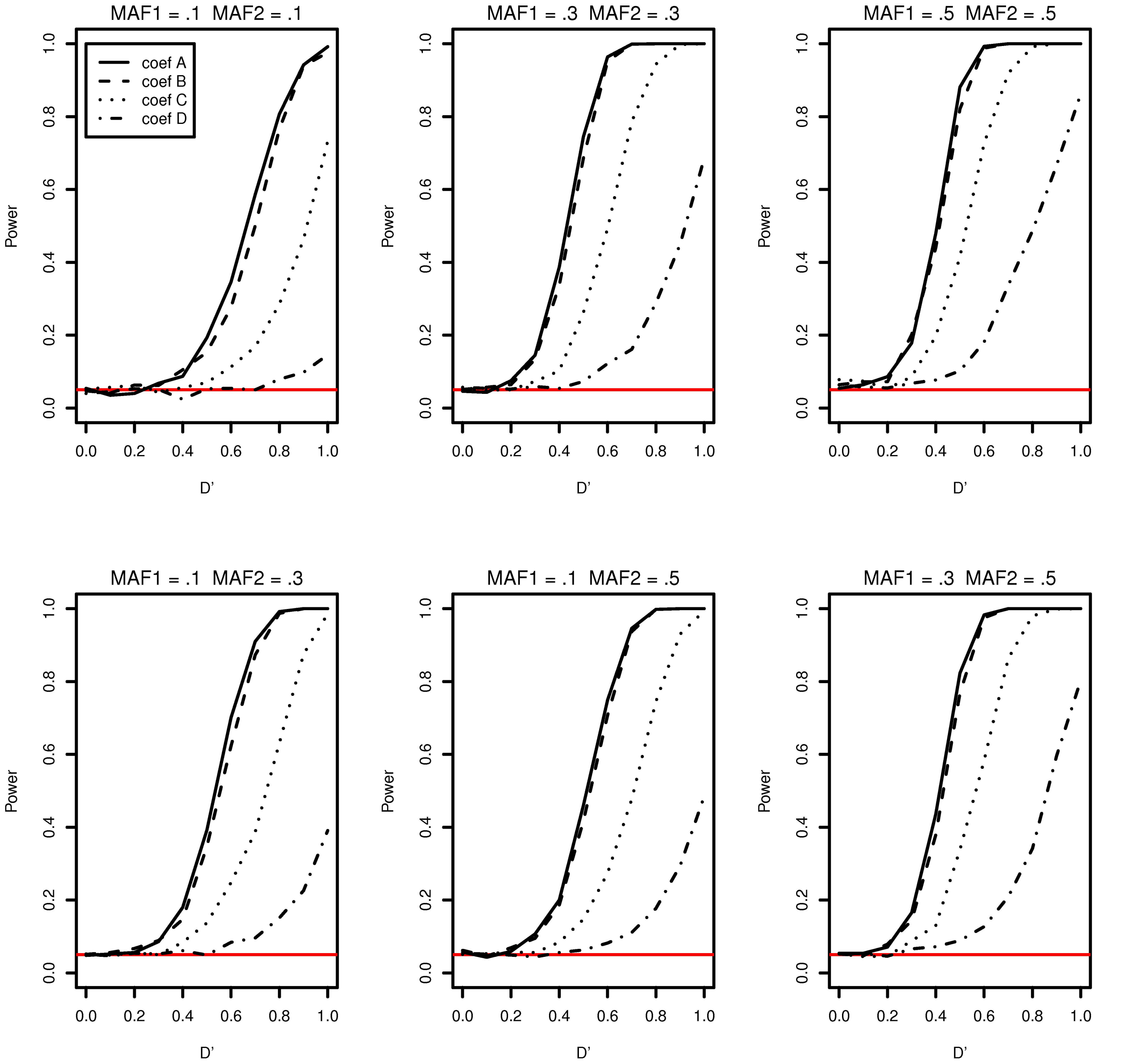

Supplemental Figure 1. Type I error and power of detecting interaction effects with different MAFs and four regression coefficients: For all four $\beta_0 = 0$, coef A: $\beta_1 = 0$, $\beta_2 = 0$, and $\beta_{12} = .5$, coef B: $\beta_1 = .25$, $\beta_2 = .25$, and $\beta_{12} = .75$, coef C: $\beta_1 = .5$, $\beta_2 = .5$, and $\beta_{12} = .5$, coef D: $\beta_1 = .75$, $\beta_2 = .75$, and $\beta_{12} = .25$, and $\sigma_a^2 = .5$ and $\sigma_e^2 = 1$, when the sample size is 5000 (2500 families). The red horizontal line indicates the .05 significance level.
